# Is the level of knowledge a predictor of rational antibiotic use in Serbia?

**DOI:** 10.1371/journal.pone.0180799

**Published:** 2017-07-10

**Authors:** Olga J. Horvat, Ana D. Tomas, Milica M. Paut Kusturica, Alisa V. Savkov, Dragica U. Bukumirić, Zdenko S. Tomić, Ana J. Sabo

**Affiliations:** 1 Department of Pharmacology, Toxicology and Clinical Pharmacology, Faculty of Medicine Novi Sad, University of Novi Sad, Novi Sad, Serbia; 2 Center for Applied Statistics, University of Novi Sad, Novi Sad, Serbia; 3 Department of Planning, Analysing and Statistics, Primary Health Care Center, Pančevo, Serbia; Kaohsiung Medical University, TAIWAN

## Abstract

**Introduction:**

The objective of this study was to investigate the knowledge, attitudes and behavior regarding antibiotics of the general population.

**Methods:**

The study sample consisted of adult subjects who consulted general practitioners at health centers in Serbia and agreed to complete the questionnaire. A total of 668 questionnaires were distributed; 500 respondents completed the entire questionnaire (response rate 74.85%).

**Results:**

The average age was 51.65 ± 16.56 years, 60.80% of the respondents were women. The median antibiotic knowledge score was 9. Predictors of adequate antibiotic knowledge were higher education level and a family member whose ocuppation was related to health-care. Overall, 58.4% of respondents believed that antibiotics could be used to treat common cold. Around a half of the respondents (47.2%) self-medicated with antibiotics at least once during their life-time, and around a quarter (24.2%) during the last treatment of infection. Patients with inadequate knowledge had 3 times higher chances of self-medicating with antibiotics compared to those with adequate knowledge. Although 98.20% of respondents claimed that antibiotic treatment should be started after a visit to a doctor and receiving a prescription, only 65.8% obtained antibiotics with prescription from a doctor during the last infection.

**Conclusions:**

Although these results demonstrate that respondents had a relatively adequate level of knowledge regarding antibiotics use, some areas of misconceptions and improper behavior were identified. Therefore, further rationalization should be focused on educational campaigns targeting the behavior of patients with regard to antibiotic use.

## Introduction

Antimicrobial resistance is a global public health problem of the 21^st^ century because bacterial resistance to antibiotics is associated with increased morbidity and mortality of serious infections and health care costs. According to the report of the National Reference Laboratory for Antimicrobial Monitoring and Surveillance, Serbia belongs to a group of European countries with the highest rates of resistance [1, 2].

Antimicrobial resistance is directly associated with improper use of antibiotics. Serbia has a long tradition of high and irrational antibiotic use, which is aggravated by self-medication with these drugs [3–5]. Prescribers’ knowledge as well as the perceptions of the general public on conditions requiring antibiotic therapy and their compliance to the treatment are essential in the control of antibiotic use and surveillance of resistance [6–8]. Therefore, most of the strategies for surveillance of antimicrobial resistance, such as guidelines, policies and educational programs, have been focused on both prescribers and the public to promote rational antibiotic use.

To launch any effective intervention in this direction, it is necessary to assess the knowledge, attitudes and behavior of the general population towards antibiotic use in a given area. However, according to available data on studies conducted so far in Serbia, they provide only a limited insight into the level of the general population awareness regarding antibiotics issues [9, 10].

Therefore, the objective of this study was to investigate the knowledge, attitudes and behavior regarding antibiotics of the general population in Novi Sad.

## Materials and methods

### Setting

The study was conducted in Novi Sad, which is the second largest city in Serbia, with 384.240 inhabitants (according to the 2011 Census). The study sample consisted of adult subjects who consulted general practitioners at four health centers in Novi Sad between November 11, 2015 and January 1, 2016, and agreed to participate in the study. After giving their written informed consent, they were instructed by the researchers on how to complete the questionnaire. The study was approved by the Ethical Committee of the Faculty of Medicine in Novi Sad and the Ethical Committee of the Health Center Novi Sad; a total of 668 questionnaires were distributed.

### The questionnaire

The questionnaire (S1 Questionnaire) used in this study is based on the questionnaire of Buke et al. [11]. Necessary modifications were made to enable correct answers to questions and statements. The content, comprehension, readability and design of the questionnaire were pre-tested on 30 adult subjects in the city of Novi Sad.

The questionnaire was divided into three sections. The first section consisted of 8 questions referring to the respondents’ socio-demographic characteristics such as age, gender, marital status, educational level, employment, number of family members in the household, number of visits to a general practitioner in the last 12 months, and having a family member working in a health care institution. The second section consisted of 12 questions to be answered “true” or “false”. They asked about the conditions requiring administration of antibiotics, how the treatment with antibiotics could be started, the length of antibiotic therapy, the meaning of “to be taken twice a day”, and whether frequent and inappropriate use of antibiotics could be harmful and dangerous. The knowledge score was determined by giving one point for each correct answer, and the maximum knowledge score was 12. The third section, which consisted of 15 yes/no questions and multiple-choice questions, was designed to determine the respondents’ attitude and behavior. These questions were related to the prophylactic use of antibiotics, self-medication with antibiotics, respondents’ reaction when they believed that the antibiotic prescribed by their doctor was ineffective, who recommended the antibiotic to them, and how they used it to treat their last infection.

To examine the indication for which respondents usually took antibiotics on their own (self-medication), they were offered an additional question to name the indication in case they had taken an antibiotic on their own. The following answers (i.e., conditions) were offered: fever, cough, sore throat, common cold, abdominal pain, infection of the skin, headache, cystitis, others. The respondents could opt for one or more than one answer.

### Data analysis

Descriptive and comparative statistical data analysis was performed with the IBM SPSS Statistics 22 (IBM Corporation, Armonk, NY, USA) software. Out of descriptive statistical methods, measures of central tendency (mean, median), measures of variability (standard deviation) and frequency were used. Two categorical variables were created–status of antibiotic knowledge (adequate/ inadequate) and self-medication (yes/no). Knowledge categories were determined according to the median of knowledge score (9), and respondents were grouped into those with adequate (score ≥9) and those with inadequate knowledge (score ≤8). Self-medication was determined based on the answers to questions in Part 3 of the questionnaire. This variable, “self-medication”, was generated by combining the answers for questions “Have you ever used antibiotics in order not to get ill” (those who answered “*yes*”) and “How did you get antibiotics during your last infection?” (those who answered any of the following–“*I used the antibiotic previously used or as advised by my friends; I used the antibiotic previously prescribed by my doctor; I asked the pharmacist and used the antibiotic recommended by him*”). The Chi-squared test was used to examine the association between categorical and socio-demographic variables. Mann-Whitney U test was used for numeric data with non-normal distribution and ordinal data. Association of respondents’ characteristics with adequate antibiotic knowledge and self-medication was first evaluated using univariate logistic regression. Multivariate logistic regression included those predictors all variables with p <0.05 in the univariate analysis. There was no multicolinearity between the predictors. Results were reported as odds ratio (ORs) with 95% confidence intervals (CIs). All p values less than 0.05 were considered significant.

## Results

### Socio-demographic characteristics

Out of 668 respondents who received the questionnaire, 159 refused to complete it, 9 did not answer all the questions, and 500 respondents completed the entire questionnaire. Thus, the response rate was 74.85%.

The average age was 51.65 ± 16.56 years, and 60.80% of the respondents were women (Table 1). Highest level of education for majority of the respondents was secondary school (50%). Most respondents had 1–3 family members. About 25% of the respondents had a family member whose occupation was related to health care.

**Table 1:** Socio-demographic characteristics of respondents.

Age (years)	n	%
>65	123	24.6
18–24	25	5.0
25–34	78	15.6
35–44	70	14.0
45–54	104	20.8
55–64	100	20.0
**Gender**		
Male	196	39.2
Female	304	60,8
**Marital status**		
Single	99	19.8
Divorced/widowed	103	20.6
Married	298	59.6
**Education level**		
Primary	38	7.6
Secondary	259	51.8
Tertiary	203	40.6
**Employment**		
Unemployed	114	22.8
Retired	135	27.0
Employed	251	50.2
**Number of family members**		
1–3	274	54.8
3–5	177	35.4
>5	49	9.8
**Number of visits to a general practitioner in the last 12 months**		
None	135	27.0
1–4	223	44.6
5–10	72	14.4
>10	70	14.0
**Having a family member working in a health care institution**		
Yes	126	25.2
No	374	74.8

### Knowledge on antibiotic use

Up to as 98.25% of respondents believed that the doctor should prescribe the antibiotic. Almost half of the respondents (45.2%) thought that antibiotic therapy could be recommended by a pharmacist (Table 2), and 58.4% thought that the antibiotics could be used to treat common cold. Overall, 96% believed that frequent and inappropriate antibiotic use was dangerous (Table 2).

**Table 2:** Knowledge on antibiotic use.

Questions	n	%
**A. Reason to use antibiotic**		
To decrease pain (T/F)	100/400	20.0
To decrease fever(T/F)	103/397	20.6
To overcome malaise and fatigue(T/F)	23/477	4.6
For common cold(T/F)	292/208	58.4
**B. Antibiotics could be started**		
With an antibiotic found at home in order not to waste time (T/F)	84/416	16.8
With prescription (T/F)	491/9	98.2
Recommended by a pharmacist (T/F)	227/273	45.4
**C. An antibiotic is used**		
Until the symptoms disappear (T/F)	217/283	43.4
Until the bottle finishes (T/F)	260/240	52.0
As advised by the doctor (T/F)	483/17	96.6
**D. When an antibiotic is to be used twice a day, it should be used after getting up in the morning and before going to bed at night** (T/F)	254/264	50.8
**E. Do you think frequent and inappropriate antibiotic use has any danger** (T/F)	480/20	96.0

T/F: true/false and percentages denote those who said “True”.

The median knowledge score of respondents was 9 (range, 2–12). Out of 500 respondents, 308 (61.6%) showed adequate knowledge, while 192 (38.4%) had inadequate knowledge. Respondents with inadequate knowledge most frequently belonged to the age group of over 65 years (35.9%), while respondents with adequate knowledge to the age group of 45–54 years (22.1%) (*p*<0.001). Statistically significant differences in the level of knowledge in relation to marital status (p<0.001) and employment (p<0.001) were recorded. Respondents with inadequate knowledge most commonly had secondary school as the highest education level (65.1%), whereas those with adequate knowledge had tertiary level of education (53.2%) (p<0.001). There was a statistically significant difference in the level of knowledge in relation to the number of visits to a general practitioner (p = 0.022). Respondents with a family member whose occupation was related to health care exhibited adequate knowledge more frequently compared to those without a family member working in a health care institution (29.9% vs. 17.7%) (p = 0.002) (Table 3).

**Table 3:** Comparison of the respondents with inadequate and adequate knowledge in relation to socio-demographic characteristics.

Variables	Inadequate knowledge	Adequate knowledge	p-value
	n = 192	n = 308	
**Age (years)**			<0.001
18–24	7 (3.6%)	18 (5.8%)	
25–34	25 (13.0%)	53 (17.2%)	
35–44	21 (10.9%)	49 (15.9%)	
45–54	36 (18.8%)	68 (22.1%)	
55–64	34 (17.7%)	66 (21.4%)	
>65	69 (35.9%)	54 (17.5%)	
**Gender**			0.145
Male	83 (43.2%)	113 (36.7%)	
Female	109 (56.8%)	195 (63.3%)	
**Marital status**			<0.001
Married	98 (51.0%)	200 (64.9%)	
Single	31 (16.1%)	68 (22.1%)	
Divorced/widowed	63 (32.8%)	40 (13.0%)	
**Education level**			<0.001
Primary	28 (14.6%)	10 (3.2%)	
Secondary	125 (65.1%)	134 (43.5%)	
Tertiary	39 (20.3%)	164 (53.2%)	
**Employment**			<0.001
Employed	72 (37.5%)	179 (58.1%)	
Unemployed	48 (25.0%)	66 (21.4%)	
Retired	72 (37.5%)	63 (20.5%)	
**Number of family members**			0.832
1–3	107 (55.7%)	167 (54.2%)	
3–5	60 (31.3%)	117 (38.0%)	
>5	25 (13.0%)	24 (7.8%)	
**Number of visits to a general practitioner in the last 12 months**			0.022
None	42 (21.9%)	93 (30.2%)	
1–4	85 (44.3%)	138 (44.8%)	
5–10	37 (19.3%)	35 (11.4%)	
>10	28 (14.6%)	42 (13.6%)	
**Having a family member working in a health care institution**	34 (17.7%)	92 (29.9%)	0.002

n–number of respondents

Six socio-demographic variables (age, marital status, education level, employment, number of visits to a general practitioner in the last 12 months and having a family member working in a health care institution) showed a significant association with the respondents’ knowledge using univariate logistic regression. These six variables were analyzed using a multivariate logistic regression to determine their independent influence on adequate antibiotic use knowledge (Table 4). The model as whole (with all predictors) was statistically significant (p<0.001). Statistically significant predictors of adequate antibiotic knowledge were higher education level (B = 1.064 p<0.001) and a family member whose occupation was related to health care (B = 0.603; p = 0.015).

**Table 4:** Multivariate logistic regression with adequate antibiotic knowledge as the dependent variable.

Independent variables	B	p	OR	95% confidence intervals	
				Lower limit	Upper limit
Age (years)	-0.047	0.646	0.96	0.78	1.16
*Marital status*					
Married	Reference category				
Single	0.151	0.636	1.16	0.62	2.18
Divorced/widowed	-0.543	0.055	0.58	0.33	1.01
Education level	1.064	<0.001	2.90	2.00	4.20
*Employment*					
Employed	Reference category				
Unemployed	-0.251	0.338	0.78	0.47	1.30
Retired	-0.343	0.271	0.71	0.39	1.31
Number of visits to a general practitioner in the last 12 months	0.108	0.364	1.11	0.88	1.41
Having a family member working in a health care institution	0.603	0.015	1.83	1.12	2.97

### Attitudes and behavior of respondents

A very low percentage of respondents reported prophylactic antibiotic use (6%) (Table 5). About 12.0% of respondents with inadequate antibiotic knowledge and 2.3% with adequate knowledge admitted that they used antibiotics for prophylaxis (p<0.001). Irregular use of antibiotics prescribed by a doctor was reported by 40% of respondents. Irregular use of antibiotics prescribed by a doctor was reported by 57.8% of respondents with inadequate antibiotic knowledge and 28.9% with adequate knowledge (p<0.001).

Almost a half of the respondents (47.2%) started antibiotic therapy on their own at least once. In the group of respondents with inadequate knowledge 63.0% and in the group of respondents with adequate knowledge 37.3% of them started antibiotics by themselves when they were ill (p<0.001).

The majority of respondents used the antibiotics prescribed by their doctor to treat their last infection (65.8%). Respondents with inadequate and adequate knowledge usually visited their doctor and used the prescribed antibiotic (46.9% versus 77.6%, respectively) (p<0.001).

About 50.6% of the respondents followed the recommendation of the doctor even if they thought that the antibiotics they were taking were not effective, although respondents with inadequate knowledge were more likely to use it for the recommended period than respondents without adequate knowledge (58.1% versus 38.5, respectively) (p<0.001).

About 39.1% of the respondents with inadequate antibiotic knowledge used antibiotics until their symptoms disappeared during their last infection, while respondents with adequate knowledge most frequently reported using antibiotics for the period advised by the doctor (76.3%) (p <0.001).

**Table 5:** Attitudes and behavior of respondents about antibiotic use.

Questions	Total n (%)	Inadequate knowledge n (%)	Adequate knowledge n (%)	p-value
A. Have you ever used antibiotics in order not to get ill?	30 (6.0%)	23 (12.0%)	7 (2.3%)	<0.001
B. Have you ever started antibiotics on your own when you got ill?	236 (47.2%)	121 (63.0%)	115 (37.3%)	<0.001
C. Have you ever used antibiotics prescribed by the doctor irregularly?	200 (40.0%)	111 (57.8%)	89 (28.9%)	<0.001
D. What do you do when you think that antibiotic you are taking is not effective?				<0.001
a) I stop taking it and go to the doctor	201 (40.2%)	88 (45.8%)	113 (36.7%)	
b) I stop taking it and go to another doctor	34 (6.8%)	26 (13.5%)	8 (2.6%)	
c) I use it for the recommended period	253 (50.6%)	74 (38.5%)	179 (58.1%)	
d) Other	12 (2.4%)	4 (2.1%)	8 (2.6%)	
E. How did you get antibiotics during your last infection?				<0.001
a) I used the antibiotic as advised by my friends or relatives	15 (3.0%)	13 (6.8%)	2 (0.6%)	
b) I used the antibiotic previously prescribed by my doctor	85 (17.0%)	53 (27.6%)	32 (10.4%)	
c) I visited my doctor and used the prescribed antibiotic	329 (65.8%)	90 (46.9%)	239 (77.6%)	
d) I asked the pharmacist and used the antibiotic recommended by him	21 (4.2%)	15 (7.8%)	6 (1.9%)	
e) I do not remember when I last used antibiotics	50 (10.0%)	21 (10.9%)	29 (9.4%)	
			
F. How did you use antibiotics during your last infection?				<0.001
a) Until the bottle is finished	108 (21.6%)	58 (30.2%)	50 (16.2%)	
b) Until the symptoms disappeared	98 (19.6%)	75 (39.1%)	23 (7.5%)	
c) As advised by the doctor	294 (58.8%)	59 (30.7%)	235 (76.3%)	

Self-medication was reported among 131 (68.2%) of respondents with inadequate knowledge and 121 (39.3%) of respondents with adequate knowledge (p<0.001).Multivariate logistic regression model includes four predictors, as shown in Table 6, which were analyzed on 500 cases out of which 252 had the defined outcome (Table 6). A test of the full model was statistically significant (p<0.001). Inadequate knowledge (B = -1,1052; p<0.001) made a significant contribution to the prediction.

**Table 6:** Multivariate logistic regression model with self-medication as a dependent variable.

Independent variable	B	p	OR	95% confidence interval	
				Upper limit	Lower limit
Age (years)	0.127	0.110	1.14	0.97	1.33
*Marital status*					
Married	Reference category				
Single	0.166	0.558	1.18	0.68	2.05
Divorced/widowed	0.186	0.497	1.20	0.70	2.06
Education level	-0.166	0.337	0.85	0.60	1.19
Inadequate knowledge	1.052	<0.001	2.86	1.91	4.29

### Reasons for self-medication with antibiotics

**Fig 1:**
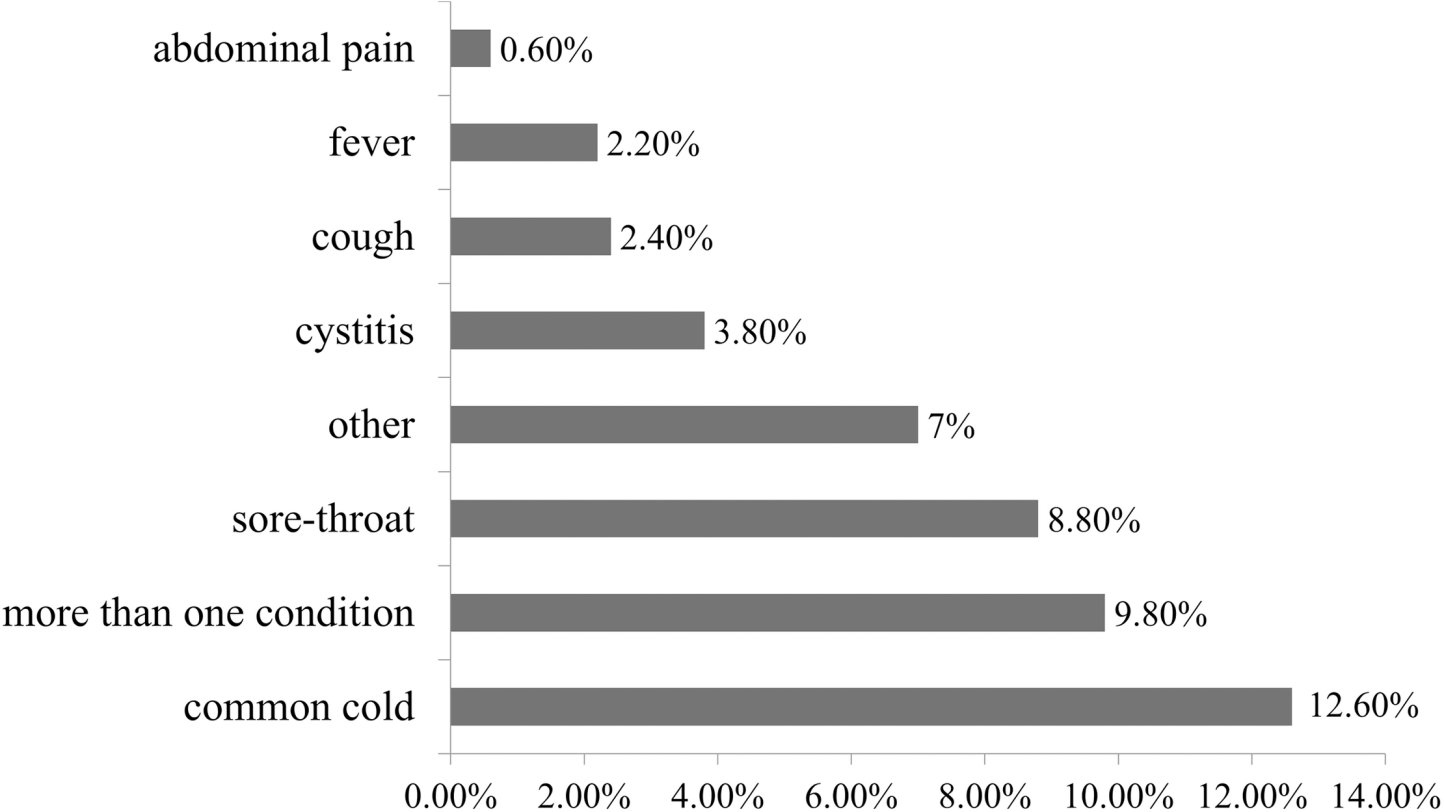
Reasons for self-medication with antibiotics.

The most common reason for self-medication was common cold (12.60%). Self-medication for more than one condition was reported by 9.80% of respondents, and some cited sore throat (8.80%) or other condition (7%) (Fig 1).

## Discussion

This study aimed to reflect the state of knowledge and behavior regarding antibiotic use of the Serbian general population, as well as to elucidate factors influencing the main outcomes of interest. To the best of our knowledge, this is the first detailed study of this type conducted in Serbia.

Despite the fact that almost two-thirds of the respondents showed adequate knowledge about antibiotics (scoring ≥9 out of 12), the study identified a number of incorrect perceptions about antibiotics and their use. For an example, more than a half of the respondents erroneously believed that antibiotics are used for common cold. Similarly, the Eurobarometer study demonstrated that the 53% of people across the EU still believe that antibiotics kill viruses and 47% believe that they are effective against colds and flu [12]. Earlier study conducted in Italy showed that majority of the respondents knew that antibiotics are not effective for common cold; however most of them believed that antibiotics can be used for sore throat, flu and fever [13]. The study performed in the United Kingdom showed that 38% did not know that antibiotics are not effective on most coughs and colds, while in Poland 38.9% of all respondents believed that antibiotics improved common cold symptoms [14, 15]. Almost a half of the respondents in our study (43%) thought that antibiotics should be stopped as soon as the symptoms resolve. This misconception of considering antibiotics as equivalent to painkillers or antipyretics has been previously reported [16–19]. Regular dosing intervals are critical for the success of the antibiotic therapy, nevertheless, in our study, half of the respondents believed that twice-daily antibiotic regimen entails taking medicine after waking up and before going to bed at night. Our results are not in agreement with the results of Buke et al. where this was noted only in 12.3% of the examined population [19].

Our study revealed two main factors significantly associated with knowledge of antibiotics—educational level and family member whose occupation was related to health care. Such findings are comparable with previously published data. The lack of knowledge among respondents with lower education has already been demonstrated in studies conducted in Italy, United Kingdom, Hong Kong, Sweden, Lithuania, Poland and Cyprus [13, 14, 20–24]. Also, positive correlation between the adequate knowledge and having a family member whose occupation was related to health care has also been reported [13,16, 25].

Besides superficiality in antibiotic knowledge of general population observed in our study, improper antibiotic behavior patterns were common among the respondents. A little less than half of participants (40%) reported using prescribed antibiotics irregularly (at least once). Likewise, almost a fifth of the respondents (19.6%) did not complete their last antibiotic course as prescribed, but stopped treatment as soon as the symptoms subsided. For comparison, Mc Nulty et al. demonstrated that more than a half of the respondents did not finish their last antibiotic course as prescribed because they felt better, which is an evidently higher value [18]. It is widely reported that irresponsible, irregular antibiotic use, inadequate dosing and incomplete courses besides leading to lack of treatment efficacy also contribute to the emergence and spread of antibiotics resistance, very current problem in Serbia [2, 26, 27].

Self-medication with antibiotics represents a common behavior that promotes resistance in one individual or among general population, with broader consequences for the global community [28]. The current finding that almost half of the study population had self-medicated with antibiotics at least once during their lifetime and that a quarter of the respondents reported self-medication during their last infection, is a share much larger than reported in other European countries [29]. The practice of self-medication of antibiotics is common worldwide, and ranges from 3% in northern Europe, over 19% in Spain, Italy and Malta, to the highest rate of 30% in eastern Europe (Poland, Latvia, Romania)[28]. In contrast to previous practice, where the major source of self-medication was buying antibiotics without prescription in private pharmacies (around 24%), in this research 17% of patients indicated that during the last treatment of infection, they used leftover antibiotics previously prescribed by a physician [4].A similar situation is noted in southern, northern, and west European countries, where leftovers are the most common source of self-medication, in contrast to Eastern European countries where purchase of antibiotics without prescription is still widely practiced [29].

In our survey, the most common reason reported by the respondents for self-medicating with antibiotics was common cold. This is probably influenced by the current treatment practice of Serbian physicians. General practitioners often prescribe antibiotics for common cold because of uncertainty whether the symptoms are caused by bacterial or viral infection (most of the antibiotics dispensed in general practice are for respiratory infections, of which more than 80% are viral in etiology) or to fulfill the patients’ demand for antibiotics [30, 31]. This misconception about antibiotics represents a significant issue for Southern and Eastern European countries (including Serbia), leading to a long tradition of high antibiotic use and, consequently, purchase of antibiotics without consulting a doctor, i.e., without prescription, which still occurs in private pharmacies in some countries [32].

Regarding the association between the knowledge and patient’s behavior in relation to antibiotic use, conflicting results have been reported in the literature. Whilst some studies found positive connection [16, 20, 33], other studies reported significant divergences [14, 11, 34]. In our study, respondents with adequate knowledge on antibiotics were more likely to report appropriate behavior with regard to antibiotic use. Respondents with adequate knowledge had significantly lower rates of prophylactic and irregular use of antibiotics, as well as lower rates of self-medication during their lifetime or during the last infection in comparison to the respondent with inadequate knowledge. The multivariate analysis demonstrated that the respondents with inadequate knowledge had almost 3 times greater odds for self-medicating with antibiotics compared to respondents with adequate knowledge. However, inadequate behavior was also documented among the population with adequate knowledge, implying certain inconsistency between knowledge and behavior. Despite the fact that 98.20% of respondents claimed that antibiotic treatment should be started after a visit to a doctor and receiving a prescription (knowledge), only 65.8% obtained antibiotics with doctor’s prescription during the last infection (behavior). This problem is not exclusive to Serbia; it was also reported in other countries, which implies the possibility of translation of study results to other regions, regardless of the sociological and other settings. This issue can be addressed through educational campaigns targeting patients’ behavior. Additionally, according to McNulty et al (2007), in order to discourage the practice antibiotic self-medication, patients must be provided with a symptomatic therapy which will fulfill the patients’ need for treatment concurrent to reducing disease signs and symptoms [18].

The strengths of the present study were the large sample size and the similarity of the sample population in terms of sex and age with the Serbian population. While efforts were made to obtain a representative sample, the overrepresentation of higher educational level (secondary and tertiary levels) in the study sample indicates selection bias. Furthermore, the individuals who were recruited for our study may have a different level of knowledge as well as attitudes towards health than the general population. Namely, as the study was conducted in an urban setting, the findings may not be generalized to the whole country. The larger-scale study is needed where more heterogeneous population mix would define further scope of antibiotic use and misuse among Serbian adults. Another limitation is that our results rely on reported rather than measured behavior. Finally, because self-administered questionnaires were used, there is a possibility that participants may have over- or under-reported socially desirable behaviors. Despite these limitations, the present study provides important findings for benefitial for public health policy makers in Serbia.

## Conclusion

Despite the fact that almost two-thirds of the respondents showed adequate knowledge about antibiotics, this study identified a number of erroneous perceptions that include thinking of antibiotics as useful in the treatment of common cold and that the treatment lasts until the symptoms disappear. Educational level and family member whose occupation was related to health care were found to be important indicators of the adequate antibiotic knowledge. Improper antibiotic behavior patterns, such as irregular use and stopping treatment as soon as the symptoms subsided, were also common among the respondents. Although respondents with adequate knowledge on antibiotics were more likely to report appropriate behavior with regard to antibiotic use, notable number of patients with adequate knowledge reported some aspect of improper behavior. Due to the noted discrepancy between patients’ knowledge and behavior regarding use of antibiotics in our study, the next step in further rationalization of the use of antibiotics should be targeted educational campaign focused on the behavior of patients in regard to antibiotic use, and constructive inclusion of physicians, pharmacists and other health care providers, who are trustworthy components of any health education and promotion programs. This kind of intervention would significantly act on the achievement of a higher degree of control of antibiotics use, but also on decrease of antibiotic resistance, which is an alarming problem in Serbia.

## Supporting information

S1 Questionnaire Questionnaire on knowledge, attitudes and behavior of patients on antibiotics use. Copy of the questionnaire used in the study, in both the original language (Serbian) and English.
(DOCX)

S1 Dataset The relevant data. (SAV)
