# Supplementary material for: Is the level of knowledge a predictor of rational antibiotic use in Serbia?
Source: PLoS One. 2017 Jul 10;12(7):e0180799. doi: 10.1371/journal.pone.0180799 (PMC5507268; doi:10.1371/journal.pone.0180799)
Supplement: S1 Questionnaire — Copy of the questionnaire used in the study, in both the original language (Serbian) and English. (DOCX) [file pone.0180799.s001.docx]

UPITNIK O POZNAVANJU, STAVOVIMA I PONAŠANJU PACIJENATA O ANTIBIOTICIMA

1. Godina rođenja? (upišite) _____________
2. Pol (zaokružite) ženski muški
3. Bračno stanje (zaokružite)
4. Udata/Oženjen
5. Neudata/Neoženjen
6. Rastavljen-a/Udovac-ica
7. Koju ste školu završili?
8. Osnovnu školu
9. Srednju školu
10. Fakultet
11. Radni status (zaokružite)
12. Zaposlen/a
13. Nezaposlen/a
14. Penzioner
15. Broj članova u domaćinstvu u kome živite
16. 1-3
17. 3-5
18. Više od 5
19. Koliko puta ste posetili lekara opšte prakse u poslednjih dvanaest meseci?
20. Niti jednom
21. 1-4
22. 5-10
23. Više od 10 puta
24. Da li je neko u vašoj porodici zdravstveni radnik?
25. Da
26. Ne

Molimo Vas da posle svake navedene tvrdnje zaokružite TAČNO ili NETAČNO

| 1. Antibiotici se koriste za snižavanje temperature | TAČNO | NETAČNO |
| --- | --- | --- |
| 1. Antibiotici se koriste protiv bolova | TAČNO | NETAČNO |
| 1. Antibiotici se koriste protiv slabosti i umora | TAČNO | NETAČNO |
| 1. Antibiotici se koriste za lečenje prehlade | TAČNO | NETAČNO |
| 1. Lečenje antibioticima se započinje antibiotikom   koji već imate kod kuće da se ne gubi vreme | TAČNO | NETAČNO |
| 1. Lečenje antibioticima se započinje nakon posete   lekaru uz dobijen lekarski recept | TAČNO | NETAČNO |
| 1. Lečenje antibioticima se započinje na   osnovu saveta farmaceuta | TAČNO | NETAČNO |
| 1. Antibiotici se uzimaju do nestanka simptoma | TAČNO | NETAČNO |
| 1. Antibiotici se uzimaju dok se ne potroši celo pakovanje | TAČNO | NETAČNO |
| 1. Antibiotici se uzimaju onoliko dugo   koliko je lekar propisao | TAČNO | NETAČNO |
| 1. Uzimanje leka dva puta na dan podrazumeva   uzimanje leka nakon buđenja  i pre odlaska na spavanje | TAČNO | NETAČNO |
| 1. Često i nepravilno uzimanje antibiotika   je štetno i opasno | TAČNO | NETAČNO |

Molimo Vas da odgovorite na sledeća pitanja:

1. Da li ste ikada uzimali antibiotik da se ne razbolite DA NE
2. Da li ste ikada neredovno koristili antibiotik koji vam je lekar propisao DA NE
3. Da li ste ikada na svoju ruku započeli uzimanje antibiotika DA NE

4. Šta radite kada mislite da antibiotik koji Vam je lekar propisao ne deluje:

1. Prestajete sa uzimanjem i odlazite ponovo kod svog lekara
2. prestajete sa uzimanjem i odlazite kod drugog lekara
3. koristite ga i dalje onako kako vam je lekar preporučio
4. ostalo (dopišite)

5. Po čijoj preporuci ste poslednji put uzimali antibiotike?

1. uzimao sam antibiotik po preporuci prijatelja, rodbine
2. uzimao sam antibiotik koji mi je već nekada ranije lekar propisao
3. uzimao sam antibiotik po preporuci lekara
4. uzimao sam antibiotik po preporuci farmaceuta
5. ne sećam se kad sam poslednji put koristio antibiotike

6. Kako ste koristili antibiotik tokom vaše prethodne infekcije:

1. Dok nisam potrošio celo pakovanje antibiotika
2. Dok nisu prestali simptomi bolesti
3. Onoliko dugo koliko mi je lekar preporučio

7. Ako ste ikada uzimali antibiotik na svoju ruku, zbog čega ste ga uzimali?

1. zbog povišene temperature
2. zbog kašlja
3. zbog bola u glu
4. usled prehlade, gripe
5. zbog bola u stomaku
6. zbog infekcije kože
7. glavobolje
8. zbog upale mokraćne bešike
9. ostalo (dopišite)

QUESTIONNAIRE ON KNOWLEDGE, ATTITUDES AND BEHAVIOR OF PATIENTS ON ANTIBIOTICS USE

1. Date of birth _____________
2. What is your gender? female male
3. What is your marritual status?
4. Married
5. Single
6. Divorced/Widowed
7. What is the highest level of school you have completed?
8. Elementary school
9. Secondary school
10. Tertiary
11. Employment status: Are you currently?
12. Employed
13. Unemployed
14. Retired
15. Number of family members in your household?
16. 1-3
17. 3-5
18. More than 5
19. How many times have you visited a general practitioner in the last 12 months?
20. None
21. 1-4
22. 5-10
23. More than 10 times
24. Is anyone in your family working in a health care institution?
25. Yes
26. No

After each statement circle TRUE or FALSE :

| 1. Reason to use antibiotic is to decrease pain | TRUE | FALSE |
| --- | --- | --- |
| 1. Reason to use antibiotic is to decrease fever | TRUE | FALSE |
| 1. Reason to use antibiotic is to overcome malaise and fatigue | TRUE | FALSE |
| 1. Reason to use antibiotic is for common cold | TRUE | FALSE |
| 1. Antibiotics could be started   with an antibiotic found at home in order not to waste time | TRUE | FALSE |
| 1. Antibiotics could be started with prescription | TRUE | FALSE |
| 7. Antibiotics could be started recommended by a pharmacist | TRUE | FALSE |
| 8. An antibiotic is used until the symptoms disappear | TRUE | FALSE |
| 9. An antibiotic is used until the bottle finishes | TRUE | FALSE |
| 10. An antibiotic is used as advised by the doctor | TRUE | FALSE |
| 11. When an antibiotic is to be used twice a day, it should be used after getting up in the morning and before going to bed at night | TRUE | FALSE |
| 12. Do you think frequent and inappropriate antibiotic use has any danger | TRUE | FALSE |

Please answer the following questions:

1. Have you ever used antibiotics in order not to get ill Yes No

2. Have you ever started antibiotics on your own when you got ill Yes No

3. Have you ever used antibiotics prescribed by the doctor irregularly Yes No

4. What do you do when you think that antibiotic you are taking is not effective?

1. I stop taking it and go to the doctor
2. I stop taking it and go to another doctor
3. I use it for the recommended period
4. Other

5. How did you get antibiotics during your last infection?

1. I used the antibiotic as advised by my friends
2. I used the antibiotic previously prescribed by my doctor
3. I visitied my doctor and used the prescribed antibiotic
4. I asked the pharmacist and used the antibiotic recommended by him
5. I do not remember when I last used antibiotic

6. How did you use antibiotics during your last infection?

1. Until the bottle is finished
2. Until the symptoms disappeared
3. As advised by the doctor

7. If you had ever started taking antibiotics on your own, for which reasons were you taking?

1. fever
2. cough
3. sore throat
4. common cold
5. abdominal pain
6. skin infections
7. headache
8. cystitis
9. other
